# Clinical and psychological phenomenology of pain in autoinflammatory diseases

**DOI:** 10.1186/s41927-020-00168-x

**Published:** 2020-12-18

**Authors:** Elisabeth Mulazzani, Nicole Zolyniak, Elisabeth Noe, Matthias Mulazzani, Shahnaz Christina Azad, Tania Kümpfel, Eduard Kraft

**Affiliations:** 1 Institute of Clinical Neuroimmunology, Biomedical Center and University Hospital, Ludwig-Maximillian University, Munich, Germany; 2 Department of Orthopaedics, Physical Medicine and Rehabilitation, Ludwig- Maximilians University, Munich, Germany; 3 Walter and Eliza Institute of Medical Research, Immunology Division, Melbourne, Australia; 4 Department of Anaesthesiology, Ludwig-Maximilian University, Munich, Germany

**Keywords:** Pain, Autoinflammatory diseases, Low penetrance variants

## Abstract

**Background:**

Pain is the clinical hallmark of patients in patients with autoinflammatory diseases (AID) caused by variants of the *NLRP3-, MEFV- or TNFRSF1A* gene. However, no systematical analysis of the clinical and psychological presentation of pain has been performed to date.

**Methods:**

Twenty-one symptomatic patients with variants in the *NLRP3*-, *MEFV*- and *TNFRSF1A* gene and clinical signs suggestive of an AID were retrospectively included in this monocentric cross-sectional case-series study. Patients were examined and interviewed using the German pain questionnaire. The hospital anxiety and depression scale (HADS) was applied to screen patients for anxiety and depression.

**Results:**

Twenty out of 21 AID patients (95%) reported pain at the time of examination. Mean current pain intensity in all AID patients comprised 3.6 ± 1.3 and mean maximum pain intensity was 7.0 ± 1.6 on a 11-point numeric ranging scale (NRS). In 15 patients (71%), pain was present for more than 60 months. Ten patients (48%) experienced recurrent attacks with asymptomatic intervals and 7 patients (33%) suffered from constant pain, while 4 patients (19%) experienced both. Nociceptive pain including musculoskeletal and visceral affection was the most prominent type of pain (*n* = 20; 95%). Pain symptoms were treated continuously with analgesic or co-analgesic drugs in 10 patients (48%). Five patients (24%) have been positively screened for concomitant depression or anxiety.

**Conclusions:**

Early and prompt diagnosis is necessary to provide multimodal pain treatment and to avoid the development of chronic pain in patients with AID.

## Background

Pain is one of the predominant characteristics in patients with autoinflammatory diseases (AID), including the cryopyrin-associated periodic syndrome (CAPS), familial Mediterranean fever (FMF) or tumor necrosis factor receptor associated periodic syndrome (TRAPS). It is also the experience of pain that significantly impacts patient’s daily life activities and drives patients with autoinflammatory syndromes to seek medical care and to use non-steroidal anti-inflammatory drugs [1]. Although the condition of pain is a diagnostic criterion in AID, only few data on clinical and psychological pain characteristics exist.

The clinical picture of AID are commonly characterized by painful flares of systemic inflammation in joints, skin, muscles, eyes and serosal surfaces, accompanied by unexplained fever and elevated acute phase reactants [2]. Involvement of the central nervous system (CNS) has also been reported [3–7] and an association with chronic demyelinating diseases was observed [8, 9].

CAPS, FMF and TRAPS are all caused by a dysregulation of the innate immune system. While CAPS is mediated via gain-of-function mutations in the *NLRP3* gene, FMF is caused by sequence variants in the *MEFV* locus encoding the inflammasome complex pyrin [10, 11]. Downstream of the inflammasome activation, both CAPS and FMF result in an overproduction of the pro-inflammatory cytokine interleukin 1β (IL-1β) [12]. In contrast, TRAPS is caused by mutations in the *TNFRSF1A* gene, which encodes the TNF receptor TNFR1. These mutations have been associated with an accumulation of mutated TNF receptors in the endoplasmic reticulum due to inadequate shedding, thus triggering an inflammatory response [13].

Prompted by the leading symptom of severe whole body-pain in one patient carrying the NLRP3 Q703K (+/−) low penetrance variant (no. 9), we planned to investigate clinical and psychological characteristics of pain in a larger cohort of AID patients. Therefore, we have consecutively investigated 21 patients with 1) clinical symptoms compatible with either CAPS, FMF or TRAPS and 2) genetically proven low penetrance variants or pathogenic mutations in the respective genes, including *NLRP3, MEFV or TNFRSF1A.*

## Methods

### Study population

Between January 2016 and March 2018, a retrospective analysis of 21 patients with clinically and genetically determined AID was conducted at the Institute of Clinical Neuroimmunology in cooperation with the interdisciplinary Pain Centre, both located within the University Hospital Munich (LMU). All patients were referred to our outpatient clinic by medical specialists including rheumatologists and neurologists. Inclusion criteria were defined as a clinical presentation suggestive for AID and the detection of low penetrance or known pathogenic mutations in the *NLRP3* (exons 4 and 6), *MEFV* (exons 2, 3 and 10) or *TNFRSF1A* (exons 2, 3, 4, and 6). Detailed demographic data including sex, age, ethnicity, underlying mutation and age at disease onset were collected. Medical data collected and evaluated include clinical manifestations, disease course, elevation of inflammatory markers (including C-reactive protein (CRP) in mg/dl, serum amyloid-A (SAA) values in mg/dl), cerebrospinal fluid (CSF) (including cell count, protein content in mg/dl, the presence of CSF specific oligoclonal bands and glucose levels in mg/dl) and magnetic resonance imaging (MRI) as well as AID-specific treatment. Written informed consent according to local ethics committee guidelines of the Ludwig-Maximilian University (project no: 600–15) and in accordance with the Declaration of Helsinki were obtained from each patient. A proportion of patients presented herein has been included in a previous clinical study [4].

### Pain assessment

Patients were structurally interviewed for the presence, intensity, location and characteristics of pain using the German pain questionnaire (DSF Deutscher Schmerzfragebogen, www.DGSS.org). Its modular approach to pain assessment consisted of demographic data, pain variables (e.g. pain localization, temporal characteristics, intensity, severity), sensory and affective qualities of pain (adjective list by Geissner, SES©), triggering and relieving factors as well as current pain treatment procedures. A numeric rating scale (NRS) scale ranging from 0 to 10 (0 = no pain, 10 = maximum conceivable pain) was used to assess patients’ subjective intensity of current, average and maximum pain intensity within the last 3 months. The affective pain quality summary score (0 = strongly disagree; 1 = disagree; 2 = agree; 3 = totally agree) was calculated by summing the responses of each item including miserable, terrible, horrible, awful. A cut-off value of ≥9 was regarded as increased affective pain experience. According to Korff [14], pain severity was graded into 4 hierarchical classes: Grade I, low disability-low intensity; Grade II, low disability-high intensity; Grade III, high disability-moderately limiting; and Grade IV, high disability-severely limiting. A 3-item inventory was used to assess the degree to which pain interferes with daily life activities, social life and occupation. Each item score ranged from 0 (no interference) to 10 (total interference). The disability score was then calculated by multiplying the mean of all three items with the factor 10. Results were classified as follows: 0–29 = 0 (no disability); 30–49 = 1 (low disability); 50–69 = 2 (moderate disability); ≥70 = 3 (high disability).

### Psychological evaluation

The Hospital Anxiety and Depression Scale (HAD-S) was used to screen AID patients for emotional distress including depressive and anxious episodes. Each item is scored on a response-scale with four alternatives ranging between 0 and 3. All responses are summed to obtain the two subscales of depression and anxiety. The most reliable cut-off values to screen successfully for mental disorders are as following: ≥ 10 on the HADS depression subscale, and ≥ 6 on the HADS anxiety subscale [15].

### Statistical analysis

Frequencies and percentages were used as descriptive statistics for categorical variables. To describe numerical variables, mean and standard deviation were used. Correlations are calculated using the Spearman’s rank correlation coefficient *r* with Prism 6.01 software (GraphPad© Software Inc., San Diego, California).

## Results

### Index patient

In 2016, a 55-year-old former employee in the field of quality management was referred to our department from the psychiatry ward because of unspecific “total body pain”. She initially sought psychiatric treatment due to her schizoaffective disorder, which began after her father’s death in 2002. At the same time, she developed persistent pain in all joints with radiation into the entire musculature. Pain qualities were described as “dragging” and “stabbing”. She had seen numerous specialists, including orthopedic surgeons, neurologists, rheumatologists and pain specialists, without receiving a definitive diagnosis. On clinical examination, she exhibited an increased sensitivity to pain over the entire body and displayed a diffuse weakness in all limbs. Maximum pain intensity was 9.0 on a 11-point numeric ranging scale (NRS, 0–10), while average pain intensity was 6.0 at the time of initial contact. A comprehensive differential diagnostic workup was undertaken. On brain MRI, multiple, supratentorial white matter lesions were present, which remained stable over time. MRI of the cervical spine showed no pathological findings. EMG and nerve conduction studies were normal. Audiometry detected a sensorineural hearing loss in the right ear, while ophthalmological examination showed a keratoconjunctivitis on both eyes. Laboratory testing repeatedly showed increased serum amyloid a (SAA) levels, but no evidence for an underlying rheumatological disease was found. Taken together, the clinical and paraclinical parameter pointed to a possible autoinflammatory condition. Genetic testing revealed a heterozygous Q703K substitution encoded by exon 3 of the *NLRP3* gene, while no other mutation was detected in *NLRP3*, *MEFV or TNFRSF1A*. Physical therapy, oral analgesics and psychotherapy partially improved her symptoms.

### Patient cohort and genetics

Consecutively, twenty-one patients (3 males, 18 females) were included in the study. All patients were Caucasian adults. The median age at study entry was 42 ± 12 years. The only NLRP3 variant found was the low penetrance substitution Q703K (*n* = 9). Aberrations in the *MEFV* gene included the pathogenic V726A (*n* = 2), M694V (*n* = 1), M680I (*n* = 1) mutations and the low penetrance substitution I591T (*n* = 2). In the *TNFRSF1A* gene, the low penetrance variant R92Q (*n* = 6; 1 homozygous (no. 16), 5 heterozygous) was exclusively found (Table 1). One patient (no. 2) carried a mutation in both *NLRP3* and *TNFRSF1A*, while in patient no. 12 no variant could be identified. Six of nine patients fulfilled the diagnostic criteria for CAPS [16], while all patients carrying MEFV pathogenic mutations or MEFV and TNFRSF1 low penetrance variants fulfilled the proposed criteria for FMF and TRAPS [17].

**Table 1:** Demographic data

	All AID	NLRP3 low penetrance variants	MEFV low penetrance variants	TNFRSF1A low penetrance variants
n	22	9	7	6
Age at study entry (y)	42 ± 12	50 ± 11	36 ± 11	42 ± 12
Sex (m/f)	3/18	1/8	2/5	2/5
Height (cm)	168 ± 9	170 ± 10	164 ± 7	165 ± 9
Weight (kg)	74 ± 17	81 ± 17	68 ± 13	71 ± 18
Ethnicity	Caucasian	Caucasian	Caucasian	Caucasian

Altogether demographic data of 21 patients with AID were assessed, one patient mutation carried mutations in the *NLRP3* and *TNFRSF1A* gene and was therefore allocated in both AID subgroups. If applicable, data are shown in absolute numbers (n), mean ± standard deviation; *y* years, *m* male, *f* female

### Clinical features

The median age at symptom onset was 28 ± 4 years. Most patients (*n* = 14; 64%) showed a recurrent disease course with flares and no intermittent symptoms. Organ involvement included the musculoskeletal system (*n* = 18; 82%), eyes (*n* = 9; 41%), skin (*n* = 12; 55%), gastrointestinal tract (*n* = 14; 64%) and CNS (*n* = 17; 77%). Of these CNS manifestations, two patients suffered from recurrent aseptic meningoencephalitis (no. 4, 5^†^), and one from cerebral vasculitis with ischemic strokes (no. 1).

Overall, 14 patients (64%) exhibited intermittently elevated levels of acute phase reactants including SAA (*n* = 14; 64%), CRP (*n* = 8; 36%) and leukocytosis (*n* = 10; 46%). Lumbar puncture was performed in 13 patients. CSF abnormalities were found in 8 patients (36%). MRI was performed in 16 patients. Ten of these patients (63%) showed abnormalities (46%), including unspecific white matter lesions as the most common neuroradiological feature.

AID-specific therapies included colchicine in 4 patients (18%) and treatment with anti-IL-1 therapies in 10 patients (46%). All detailed clinical data are comprehensively summarized in Table 2.

**Table 2:** Clinical features of AID patient cohort

	All	NLRP3 low penetrance variants	MEFV low penetrance variants	TNFRSF1A low penetrance variants
Disease course				
-Recurrent	14 (64%)	7 (32%)	5 (23%)	2 (9%)
-Chronic	0	0	0	0
-Chronic with flares	8 (36%)	4 (18%)	2 (9%)	2 (9%)
Age at disease onset (y)	28 ± 4	36 ± 4	18 ± 3	31 ± 5
Diagnostic latency (y)	11 ± 4	8 ± 2	14 ± 3	12 ± 7
Positive family history	14 (64%)	4 (18%)	6 (27%)	4 (18%)
Organ involvement				
-Musculoskeletal	18 (82%)	7 (32%)	5 (23%)	6 (27%)
-Eyes	9 (41%)	5 (23%)	2 (9%)	2 (9%)
-Skin	12 (55%)	5 (23%)	2 (9%)	5 (23%)
-Gastrointestinal tract	14 (64%)	4 (18%)	5 (23%)	5 (23%)
-CNS	17 (77%)	8 (36%)	4 (18%)	5 (23%)
Constitutional symptoms				
-Fatigue	13 (59%)	7 (32%)	2 (9%)	4 (18%)
-Fever	12 (55%)	4 (18%)	4 (18%)	4 (18%)
Elevation of inflammatory markers				
-SAA	14 (64%)	6 (27%)	4 (18%)	4 (18%)
-CRP	8 (36%)	2 (9%)	5 (23%)	1 (5%)
-Leukocytosis	10 (46%)	6 (27%)	2 (9%)	2 (9%)
CSF (*n* = 11)				
-Abnormal	8 (36%)	5 (23%)	1 (5%)	2 (9%)
MRI (*n* = 16)				
-Abnormal	10 (46%)	7 (32%)	1 (5%)	2 (9%)
AID specific treatment				
-Colchicine	4 (18%)	0 (0%)	3 (14%)	1 (5%)
-Anti IL-1 therapy	10 (46%)	6 (27%)	1 (5%)	3 (14%)

### Pain characteristics

**Fig. 1:**
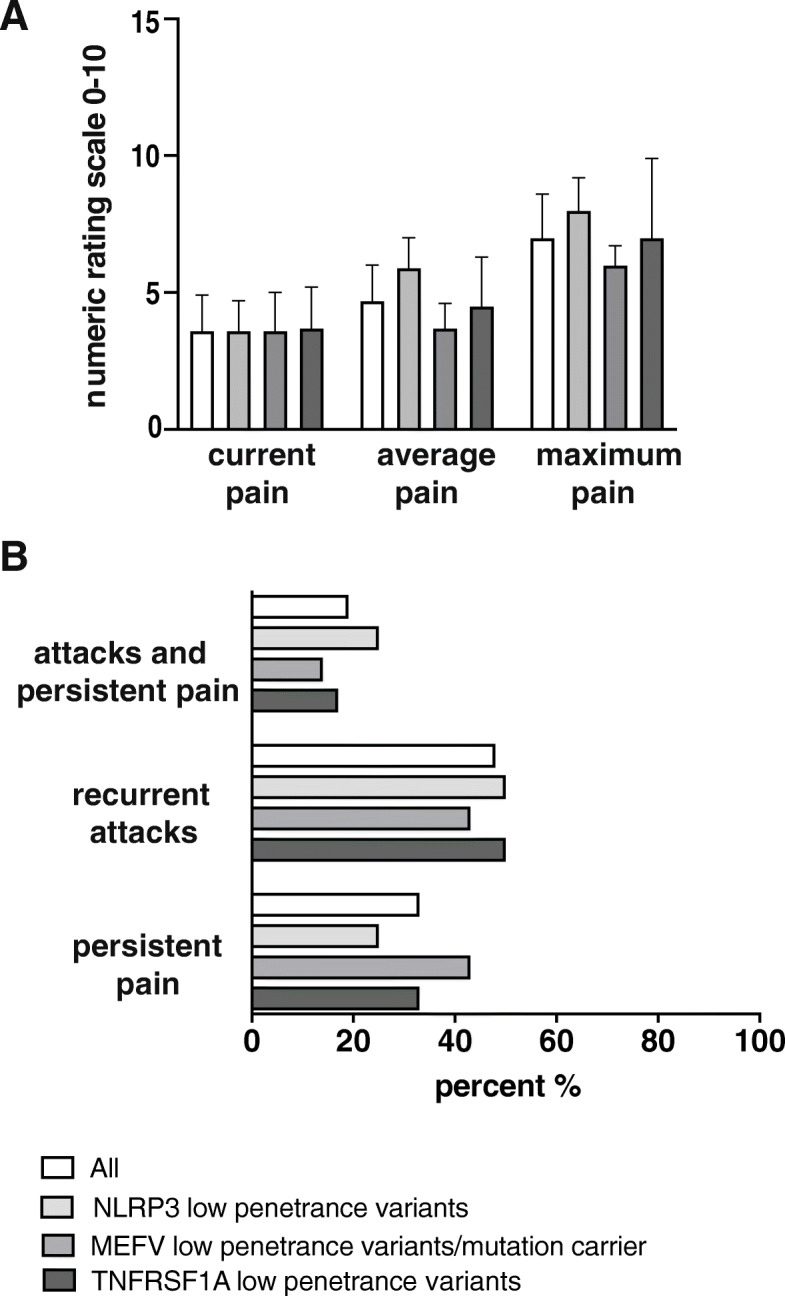
Pain intensity and temporal aspect of pain. **a** Pain intensity including current, average and maximum pain levels of either total AID patients or *NLRP3*, *MEFV* and *TNFRSF1A* low penetrance variants alone are plotted on a numeric ranging scale (0 = no pain, 10 = maximum conceivable pain). Error bars indicate standard error of the mean. White columns: all patients; light grey columns: *NLRP3*; grey columns: *MEFV*, and dark grey columns: *TNFRSF1A* low penetrance variants. **b** Pain episodes of all patients (white columns), *NLRP3* (light grey columns), *MEFV* (grey columns) and *TNFRSF1A* (dark grey columns) low penetrance variants alone are categorized as follows: persistent pain, recurrent attacks with asymptomatic intervals or attacks with persistent pain in between. Data are shown as relative numbers in percent (%)

**Fig. 2:**
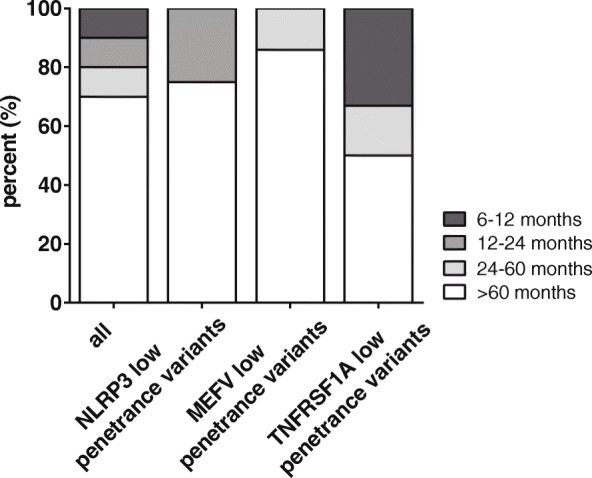
Pain duration. Pain duration ranging from either 6–12 (dark grey box), 12–24 (grey box), 24–60 (light grey box) or more than 60 months (white box) of total AID patients, *NLRP3*, *MEFV* and *TNFRSF1A* low penetrance variants alone are shown. Data are presented as relative numbers in percent (%)

Twenty out of 21 AID patients (95%) reported ongoing pain at the time of examination. Mean current pain intensity in all AID patients was 3.6 ± 1.3) on the 11-point numeric ranging scale (NRS) from 0 to 10. Mean maximum pain intensity was 7.0, ranging from 5.4 to 8.6 on NRS. Mean average pain intensity was 4.8 ± 1.2) at the time of examination. Average values for current, average and maximum pain intensity are shown in Fig. 1a. Ten patients experienced recurrent attacks with asymptomatic intervals (48%), while 7 patients suffered from constant pain (33%). Four patients (19%) suffered from recurrent pain exacerbations with persisting pain (Fig. 1b). In 15 patients (70%), pain was present for more than 60 months (Fig. 2).

Pain was located most commonly in the legs (*n* = 13; 62%), arms (*n* = 10; 48%), shoulders (*n* = 9; 43%), hands (*n* = 9; 43%) and head (*n* = 9; 43%), while chest pain was only reported in 3 AID patients (14%). The vast majority of patients (*n* = 18; 86%) reported pain in more than two body regions. Eighteen out of 21 total AID patients complained about ≥2 different pain qualities (86%). The most frequently reported pain qualities were “dragging” (*n* = 17; 81%), “stabbing” (*n* = 13; 62%) and “dull” (*n* = 12; 57%). With the exception of patient no. 1, all patients displayed nociceptive pain including musculoskeletal and visceral pain affection. Only patient no. 1 showed an intermittent neuropathic pain episode with painful dysesthesias in the lower limbs due to a corresponding lesion in the thoracic spinal cord on level 12 five years earlier, which recovered completely during the following months. Detailed pain localizations and sensory qualities of each individual patient are presented in Table 3.

**Table 3:** Pain characteristics

Patient number/age/sex/origin	Low penetrance variants/mutations	Localization of pain	Sensory qualities of pain	Affective pain quality summary score	Pain trigger/ intensifying factors	Pain relieving factors	Pain severity according to Korff	Dis-ability score	Dis-ability days	De-pression score	Anxiety score	Actual analgetic therapy
**1**/68/F/C	Q703K (+/−) *NLRP3* gene (exon 3)	–	–	–	–	–	–	0	0	13	6	none
**2**/52/F/C	Q703K (+/−) *NLRP3* gene (exon 3); R92Q (+/−) *TNFRSF1A* gene (exon 4)	arms, legs, abdominal	dragging, burning	1	bacterial/viral infection, mental stress, injury	relaxation, yoga, physiotherapy	I	0	10	1	6	ibuprofen, diclofenac, acetylsalicylic acid, paracetamol
**3**/60/F/C	Q703K (+/−) *NLRP3* gene (exon 3)	head, thoracic, lower back, shoulder, arms, hands, legs, feet, abdominal	dull, stabbing, dragging, burning	9	mental stress	take a walk, sleeping	IV	3	8	10	16	ibuprofen, steroids
**4**/33/M/C	Q703K (+/−) *NLRP3* gene (exon 3)	head, cervical, shoulder	dull, stabbing, dragging	6	coldness, mental stress	relaxation, stretching	III	3	5	3	7	none
**5**/63/F/C^†^	Q703K (+/−) *NLRP3* gene (exon 3)	lower back, shoulder, pelvic area, legs, feet,	pulsatile, throbbing, stabbing, dragging, burning	4	n.k.	physio-, ergo-therapy, sleeping, exercising	IV	3	90	8	11	none
**6**/52/F/C	Q703K (+/−) *NLRP3* gene (exon 3)	head, lower back, shoulder, arms, hands, pelvic area, legs, feet	dull, dragging	6	mental stress	exercising, stress prevention, sleep, relaxation	III	1	12	3	4	none
**7**/40/F/C	Q703K (+/−) *NLRP3* gene (exon 3)	pelvic area	dragging	1	fatigue, mental stress	relaxation, medication	I	0	0.5	11	8	tramadol, ibuprofen, paracetamol, diclofenac
**8**/47/F/C	Q703K (+/−) *NLRP3* gene (exon 3)	head, cervical, thoracic, lower back, shoulder, pelvic area, legs	dull, dragging, burning	1	coldness, physical stress	sleep, exercising, change in climate	IV	2	30	3	5	none
**9**/59/F/C	Q703K (+/−) *NLRP3* gene (exon 3)	head, shoulder, arms, hands, pelvic area, legs, feet	stabbing, dragging	6	coldness	no influence-able factors are known	IV	3	20	19	12	ibuprofen
**10**/29/M/C	V726A (+/−) *MEFV* gene (exon 10)	head, cervical, abdominal	dragging	7	stress	tranquility, mediaction	III	1	10	2	3	none
**11**/49/F/C	I591T (+/−) *MEFV* gene (exon 9)	head, hands, abdominal	dull	5	food	nutrition, tranquility, warmness	I	0	0	2	7	ibuprofen
**12**/23/F/C	unknown	cervical, shoulder, chest	dull, pulsatile, stabbing	9	over-exercising, stress, sleep deprivation	sleep	I	1	14	2	2	none
**13**/43/F/C	M694V (+/−) *MEFV* gene (exon 10)	hands, legs, abdominal, chest	pulsatile, stabbing, dragging, burning	7	stress	sleep	IV	3	21	19	16	ibuprofen
**14**/39/M/C	V726A (+/−) *MEFV* gene (exon 10)	abdominal	dull, pulsatile, stabbing, dragging, burning	12	n.k.	no influenceable factors are known	IV	3	12	11	7	tramadol, tilidine, oxycodone, etoricoxib
**15**/33/F/C	I591T (+/−) *MEFV* gene (exon 9)	head, cervical, thoracic, lower back, shoulder, arms, hands, pelvic area, legs, feet, abdominal, chest	dull, dragging, burning	7	over-exercising, stress	meditation, reading, take a walk, meeting friends	IV	2	40	3	5	none
**16**/55/F/C	M680I (+/−) *MEFV* gene (exon 10)	thoracic, shoulder, arms, hands, pelvic area, legs, feet	dull, stabbing, dragging	9	stress	exercise, psychotherapy	IV	2	45	9	12	none
**17**/32/F/C	R92Q (+/−) *TNFRSF1A* gene (exon 4)	head, cervical, shoulder, arms, legs	dull, pulsatile, stabbing, dragging	5	fatigue, stress	take a walk, sleep	III	0	42	6	8	ibuprofen
**18**/55/F/C	R92Q (+/+) *TNFRSF1A* gene (exon 4)	hands, feet, abdominal	dull, stabbing, dragging	4	warmness	take a walk, breathing exercises, distraction	II	1	0	9	9	none
**19**/43/F/C	R92Q (+/−) *TNFRSF1A* gene (exon 4)	thoracic, lower back, arms, hands, legs, feet abdominal	dull, stabbing, dragging, burning	2	n.k.	tranquility, relaxation, medications	III	2	0	7	0	none
**20**/31/F/C	R92Q (+/−) *TNFRSF1A* gene (exon 4)	thoracic, arms, hands, pelvic area, legs, feet, abdominal	stabbing, dragging	6	weather changes, food intake, mental stress	distraction, meditation, exercise	IV	2	25	5	5	steroids
**21**/56/F/C	R92Q (+/−) *TNFRSF1A* gene (exon 4)	legs	stabbing, pruritic	1	warmness, alcohol, period, stress	cold, fresh air, sleep, relaxation, distraction	I	1	0	7	5	morphin, tramadol, pregabaline

*f* female, *m* male, *C* Caucasian

The most frequent self-reported trigger of pain flares was stress (*n* = 14; 67%), while the most commonly mentioned pain-relieving factor was physical exercise (Table 3).

According to Korff and collegues [14], pain severity was classified as “IV = severe” in 9 AID patients (43%).

Pain symptoms were treated continuously with analgesic or co-analgesic drugs in 10 of 21 patients (48%), while half of them were prescribed more than one analgesic substance. Eight patients received nonsteroidal anti-inflammatory drugs (NSAID). In two patients (no. 3 and 20) steroids were administered, while in one patient (no. 21) a calcium channel blocker was administered to reduce pruritic pain (Table 3). Three patients were treated with opioids (no. 7, 14, 21).

### Depression, anxiety and pain impact on daily life

An increased affective pain quality score (cut-off ≥9) was found in 4 patients (19%). In the total patient cohort, the mean depression sub score (HADS-D) was 7.3 ± 1.1), while mean anxiety sub score (HADS-A) was 7.3 ± 0.9). Five patients (24%) scored ≥11 in the depression test scale (HADS-D; cut-off ≥10) suggesting a possible depressive disorder, while also five patients (24%) had a score of ≥11 in the anxiety test scale (HADS-A; cut-off ≥6) pointing to the potential cooccurrence of anxiety [15]. Self-reported disability days ranged from zero to 90 days within the last 3 months. Disability score of six patients with AID (29%) was at the highest level “3”.

**Fig. 3:**
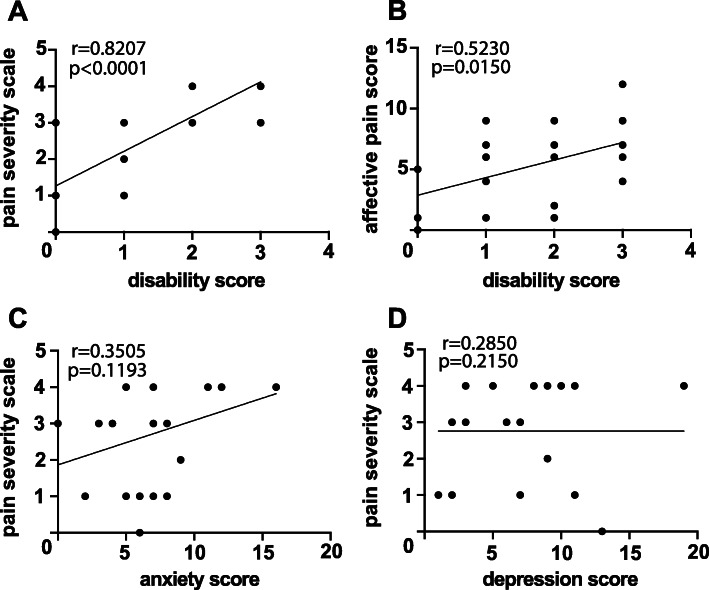
Relationship between physical and psychological pain characteristics. Correlations of indicated pain scores are shown: **a** pain severity score versus disability score, **b** affective pain score versus disability score, **c** pain severity score versus anxiety and **d** pain severity score versus depression. Spearman’s correlation coefficients (r) and *p*-values (p) are reported. A linear trendline is shown

Spearman’s rank correlation was calculated to determine the relationship between pain severity and disability (*r* = 0.8207; *p* < 0.0001) (Fig. 3a), affective pain score and patients’ disability (*r* = 0.5230; *p* = 0.0150) (Fig. 3b), pain severity and anxiety (*r* = 0.3505; *p* = 0.1193) (Fig. 3c) as well as pain severity and depression (*r* = 0.2850; *p* = 0.2150) (Fig. 3d).

## Discussions

Autoinflammatory syndromes are rare, debilitating diseases and demand interdisciplinary treatment strategies due to multi-organ involvement. Although painful flares of skin, eyes, joints, muscles and CNS are part of the diagnostic criteria in AID [16, 18, 19], the pattern of pain and it’s contributing psychological factors often remain neglected and detailed structured parameters are still missing.

Our index patient initially presented with “whole body pain”, which persisted and generalized to the point of causing significant disability for more than 10 years. Extensive evaluation by numerous specialists had been unrevealing. The diffuse nature of her pain together with psychiatric comorbidities aggravated finding the definitive diagnosis. Intrigued by this case, we subsequently set out to systemically review the pain patterns and the impact of psychological factors in patients with low penetrance variants in the *NLRP3*, *MEFV* or *TNFRSF1A*. In our patient cohort, pain was present at a very high frequency, most commonly of nociceptive nature, following a chronic course and presenting as recurrent attacks and persisting pain alike. Clinical pain presentation was heterogenous and a distinct phenotype could not be attributed to a certain autoinflammatory syndrome. While other groups have found abdominal pain to be present in 95% of all FMF patients and have described an increased frequency of *MEFV* mutations in patients with epigastric pain syndrome [20–22], in our patient cohort, abdominal pain was only present in 71% of *MEFV* low penetrance variants. This finding may be partially explained by the low frequency of pathogenic mutation carriers in our study.

The underlying mechanism for pain manifestation in AID patients is poorly understood. As almost all patients displayed nociceptive pain including musculoskeletal and visceral pain affection, an inflammatory nature of pain seems likely as patients with AID show a hyperinflammatory phenotype with increased levels of IL-1β, IL-6 and TNF-α following in vitro PBMC stimulation [23, 24]. The cytokine TNF-α itself serves as a key regulator during the inflammatory cascade and triggers the production of pro-algetic agents. Consecutively, etanercept, a TNFα-inhibitor, has been successfully used in the treatment of rheumatoid arthritis and other chronic inflammatory diseases [25]. A more modest reduction of inflammation has been observed following anti IL-1 therapy [26]. Furthermore, IL-6 knockout mice showed reduced mechanical and thermal hyperalgesia in response to inflammatory stimuli [27]. However, IL-1β, IL-6 and TNF-α may also act directly on nociceptors or stimulate the release of prostaglandins and therefore perpetuate inflammation [28]. Interestingly, in our cohort, AID-specific treatment led to a reduced prescription of analgesic or co-analgesic drugs, underscoring the possible inflammatory nature of pain manifestation in AID.

Notably, chronic pain is one of the critical factors for determining depression, and their coexistence tends to further aggravate and perpetuate the severity of both disorders. In our patient cohort, 24% of the study population were positively screened for depression and anxiety. Self-reported average working capacity and social life activities were significantly impaired due to pain manifestations. Here, the traditional explanatory causation model, whereby pain and disability induced by physical illness leads to mental health problems, becomes replaced by a more complex, integrated view on pathophysiology. In rheumatoid arthritis, for example, emerging data illustrate the negative effects of proinflammatory cytokines on monoaminergic neurotransmission, neurotrophic factors, and measures of synaptic plasticity. Accumulating evidence includes findings from clinical trials of immunomodulatory therapy, indicating that these interventions can provide benefits to mental health independent of improvements in physical disease scores [29].

Patients with low penetrance variants have been reported to exhibit a milder phenotype, usually lacking both CNS symptoms and an up-regulation of the IL-1β axis [30–32]. In contrast to these findings, we have described several patients with low penetrance variants and severe CNS manifestations [4, 6, 7]. Given the heterogeneous clinical phenotype and their variable penetrance, these low penetrance variants may exert their proinflammatory effects in combination with environmental or other genetic susceptibility factors. Taken together, the clinical and functional significance of the low penetrance variants remains a matter of debate [4, 33, 34]. Therefore, our study is limited by the fact that the majority of patients carried low penetrance variants, while only four patients displayed known pathogenic mutations, all located in the *MEFV* gene. Further and more sophisticated genetic investigations such as whole-exome sequencing and the exclusion of somatic mosaicism is desirable to detect yet unknown mutations. A further limitation is the relatively small sample size. Accordingly, a multi-centre study with larger number of patients is desirable to confirm our results.

As has been demonstrated by our index patient, it is common for rare AID patients to report unusual symptoms. As such, patients are frequently mislabelled as “hypochondriacs”, or as having a psychosomatic disorder. As a consequence, AID has to be considered in patients with heterogenous pain syndromes to effectively diagnose, treat and ultimately prevent patients from the development of chronic pain and the loss of functionality in their working and social life. Accordingly, we strongly recommend the routine use of supplementary questionnaires and patient-reported outcome measures amending the clinical status in a multidisciplinary setting [35].

Taken together, our data highlight the complexity in assessment and management of pain and emphasizes the necessity of an integrated biopsychosocial view on AID as well as on other rheumatic diseases.

## Conclusions

Monogenetic autoinflammatory diseases (AID) are rare and debilitating diseases, caused by a dysregulation of the innate immune system. In patients with AID, pain occurs at a high frequency, regardless of the underlying genotype. Therefore, these patients will be encountered by pain specialists in clinical practice. Furthermore, pain in AID patients carrying low penetrance variants is of a nociceptive nature and often causes significant disability in social and working life. Contrary to widespread believe, affective components play an inferior role in pain perpetuation among these patients.

## Data Availability

All data generated or analyzed during this study are included in this published article.

## Abbreviations

AID: Autoinflammatory diseases
CAPS: Cryopyrin-associated periodic syndromes
FMF: Familial Mediterranean fever
NRS: Numeric ranging scale
TRAPS: Tumor necrosis factor receptor 1-associated periodic syndrome
